# A Shocking Case of Pacemaker Lead Perforation

**DOI:** 10.1016/j.jaccas.2022.07.003

**Published:** 2022-09-21

**Authors:** Eli Simsolo, Bruce L. Wilkoff

**Affiliations:** a Division of Cardiology, Tufts Medical Center, Boston, Massachusetts, USA; b Section of Cardiac Pacing and Electrophysiology, Heart and Vascular Institute, Cleveland Clinic Foundation, Cleveland, Ohio, USA

**Keywords:** chest pain, pacemaker lead dysfunction, perforation, CT, computed tomography, ICD, implantable cardioverter-defibrillator, RA, right atrial, RV, right ventricular, TEE, transesophageal echocardiogram

## Abstract

We report on a 66-year-old man who presented with presyncope, chest discomfort, and pectoralis muscle stimulation after pacemaker implantation. Imaging confirmed lead perforation through the myocardium and reaching the anterior chest wall. (**Level of Difficulty: Intermediate.**)

## Central Illustration

**Figure:**
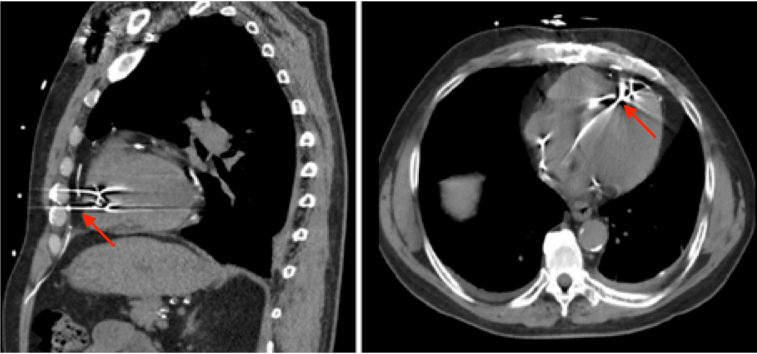

## History of Presentation

A 66-year-old man presented to the cardiology clinic for a second opinion. On presentation, he had mild intermittent presyncope and left-sided chest discomfort at rest and with exertion that started several months after implantation of his dual-chamber pacemaker.

**Learning Objectives**

• To be attentive to the complications that can result from lead perforation.
• To understand the risks and benefits of lead retrieval vs abandonment in the setting of acute and chronic lead perforation.

## Past Medical History

Outside records revealed a past medical history significant for coronary artery disease for which he underwent percutaneous coronary intervention and coronary artery bypass graft surgery, as well as dual-chamber pacemaker implantation for complete heart block and asystole 2 years earlier. The patient underwent chest computed tomography (CT) at an outside facility after he presented with chest wall stimulation. Chest CT demonstrated right ventricular (RV) lead perforation through the epicardial surface that was causing stimulation of the pectoralis muscle. The perforated lead was capped and abandoned, and a new RV lead was placed 21 months after the initial device implantation.

## Differential Diagnosis

The differential diagnosis included acute coronary syndrome and pacemaker malfunction.

## Investigations

Device interrogation revealed that his ventricular capture thresholds had increased. Left-sided heart catheterization revealed widely patent drug-eluting stents and vein grafts.

Physical examination from his initial presentation revealed rhythmic contractions of his left pectoralis muscle (Video 1).

**Figure 1:**
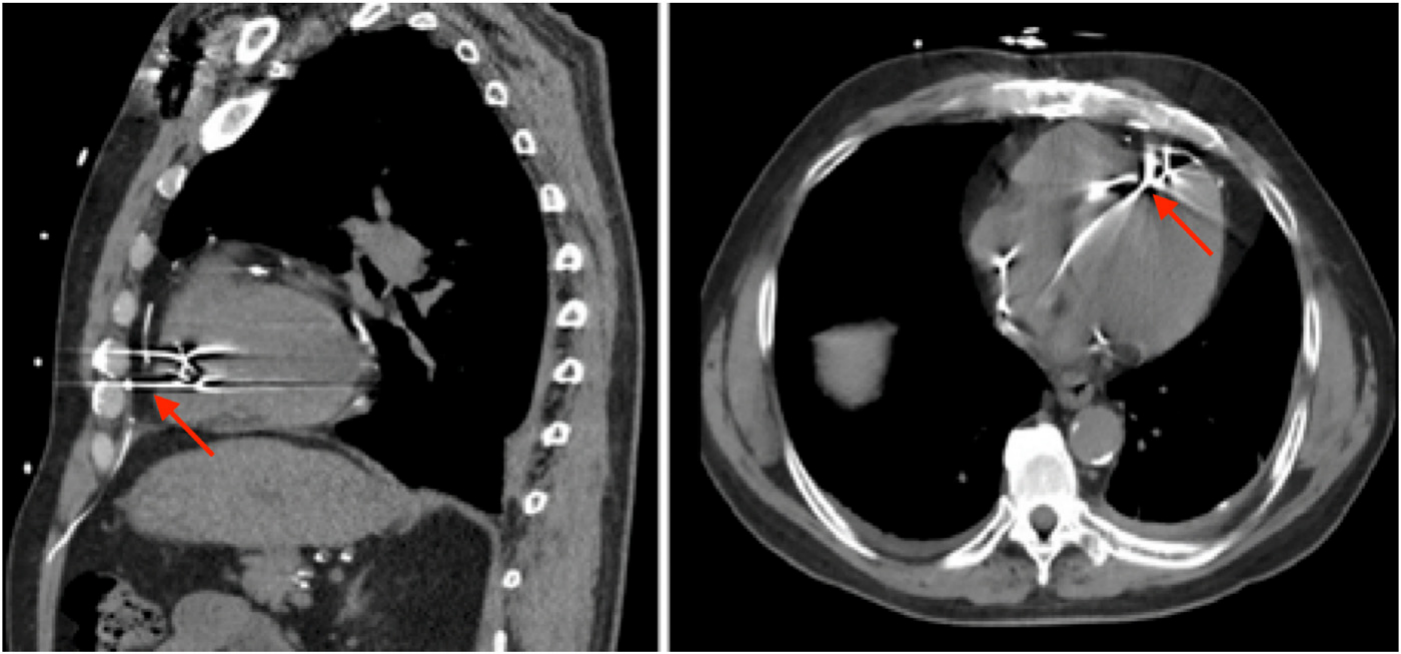
Chest Computed Tomography of Pacemaker Lead Perforation The images display a lead tip perforating the right ventricular myocardium and pericardium **(red arrows).**

Imaging suggested that the abandoned RV lead had perforated the epicardial surface anteriorly, had reached the anterior chest wall, and was causing pectoralis muscle stimulation (Figure 1).

## Management

The patient presented to our clinic for a second opinion on lead removal, and he subsequently underwent successful percutaneous extraction. The active RV lead was found to be functioning normally; however, the right atrial (RA) lead demonstrated outer insulation damage and high capture thresholds. Both the RA lead and the capped RV lead were removed with a locking stylet and a 14-F laser sheath. The capped RV lead slid back easily once the laser was advanced under the clavicle. A transesophageal echocardiogram (TEE) was used to rule out pericardial bleeding after the perforating RV lead was extracted, and a new RA lead was inserted.

## Discussion

Lead perforation of the myocardium and migration of the tip of the lead to extracardiac structures comprise an infrequent but significant complication of transvenous pacing and implantable cardioverter-defibrillator (ICD) leads. The incidence of this major complication varies from 0.1% to 1% depending on the device implanted, permanent pacemaker or ICD.^1^, ^2^, ^3^ Delayed lead perforations (those diagnosed at least 1 month after implantation) are rarer than acute lead perforations. However, 1 study showed that the prevalence may be underestimated, and it may be significantly higher among asymptomatic patients when evaluating these patients with CT scans.^4^

Mechanical migration of a lead can result in pericardial inflammation, as well as pericardial effusion and tamponade. In addition, the lead tip can enter the pleural space, pulmonary parenchyma, or the abdomen and viscera. Complications such as pleural effusions, pneumothorax, and hemothorax may be a consequence of vascular and cardiac injury. The electrical consequences are poor sensing, poor capture, and extracardiac stimulation, including intercostal stimulation, diaphragmatic stimulation, and, as in this case, pectoral muscle stimulation. Depending on the complication from the lead migration, patients may present with coughing, pain, hemoptysis, shortness of breath, or no symptoms at all.

Several risk factors have been shown to increase the incidence of acute, subacute, and delayed lead perforation, including: female sex, decreased diameter of the lead, age >80 years, lead tip stiffness, and lead location. Lead placement on the atrial or ventricular septal walls may carry a decreased risk of perforation.^5^^,^^6^ The type of lead migration complications that arise also depends on the timing of implantation, the lead location (RA vs RV), and the device type (ICD vs permanent pacemaker).^7^

The question of management, either abandoning or removing an inactive lead, typically depends on the presence or absence of symptoms, the current and future risk of leaving the lead in place, and the risk of removal. Some clinicians argue that chronically perforated leads that have not resulted in complications to the patient or the device do not necessitate removal.^4^ However, patients with device malfunction or patients with symptoms require lead extraction either surgically or percutaneously. As demonstrated in this case, the decision to cap and abandon the initial RV lead did not alleviate the patient’s symptoms, although the pectoralis muscle stimulation had resolved. He continued to report chest discomfort and presyncope. In his case, percutaneous lead removal was easily accomplished without complications, and is the most common outcome. However, given the potential for active bleeding and hemodynamic collapse, it is essential that lead removal should occur in a setting where the patient can be adequately monitored and rescued when required. Specifically, the procedure should be performed in an operating room with close monitoring under TEE and a cardiac surgical team in case of hemodynamic collapse.^8^ In the case of hemodynamic instability, other visceral injuries, or cardiac tamponade, a surgical approach would be more appropriate.^9^^,^^10^ Our examination and imaging studies did not demonstrate evidence of visceral damage or cardiac tamponade, and therefore, a percutaneous approach was deemed a suitable initial approach.

Furthermore, given that veins can accommodate only a finite number of leads, serious consideration should be given to removing nonfunctioning leads because they are more difficult to extract after fibrotic tissue has built up over time. In view of the patient’s relatively young age and robust functional capacity, we predicted that he may require a generator change and lead exchanges in the future. As previously noted, his RA lead demonstrated outer insulation damage and high capture thresholds requiring lead exchange at the time of our intervention.^11^

## Follow-Up

Since extraction and implantation of his new RA lead, the patient has been doing well. Device interrogation after the procedure exhibited that capture, sensing, and lead impedances were appropriate.

## Conclusions

Patients presenting with pectoral muscle stimulation secondary to lead perforation are rare, but this case demonstrates the need to look for the root cause of unusual phenomena. The question whether to retrieve leads in the case of delayed perforation should be tailored to the patient’s presentation (symptoms, hemodynamic stability) and centered around an informed discussion with the patient.

## Funding Support and Author Disclosures

Dr Wilkoff has served as a consultant for Abbott, Philips, and Medtronic. Dr Simsolo has reported that he has no relationships relevant to the contents of this paper to disclose.

## Appendix

Supplemental Video 1 Rhythmic Contractions of the Patient’s Left Pectoralis Muscle
